# Choroidal structural and perfusion characteristics across refractive groups in children

**DOI:** 10.3389/fmed.2026.1861658

**Published:** 2026-06-10

**Authors:** Yu Liu, Getu Tao, Yifan Zhao, Mengyao Ma, Shuang Feng, Min Qin, Xiuli Bao

**Affiliations:** 1 Department of Ophthalmology, The Affiliated Hospital of Inner Mongolia Medical University, Hohhot, China; 2 School of Public Health, Inner Mongolia Medical University, Hohhot, China

**Keywords:** childhood myopia, choroid, choroidal vascularity index, Haller layer, optical coherence tomography, optical coherence tomography angiography

## Abstract

**Objective:**

To characterize choroidal structural and perfusion changes in children with emmetropia, low myopia, and moderate myopia using enhanced depth imaging optical coherence tomography (EDI-OCT) and optical coherence tomography angiography (OCTA), and to examine their associations with spherical equivalent refraction (SER) and axial length (AL).

**Methods:**

In this retrospective cross-sectional study, the clinical records and OCT/OCTA images of 140 children aged 8–14 years (140 right eyes) examined at the Ophthalmology Outpatient Department of the Affiliated Hospital of Inner Mongolia Medical University between October 2024 and October 2025 were reviewed. Participants were classified into emmetropia (30 eyes), low myopia (70 eyes), and moderate myopia (40 eyes) groups according to cycloplegic SER. EDI-OCT was used to measure choroidal thickness (CT), Haller layer thickness (HLT), Sattler’s layer–choriocapillaris complex thickness (SLCCT), stromal area (SA), luminal area (LA), total choroidal area (TCA), and choroidal vascularity index (CVI). OCTA was used to quantify full choroidal perfusion density (FCPD), choriocapillaris perfusion ratio (CCPR), choriocapillaris flow-deficit percentage (CCFD), number of choriocapillaris flow deficits (CCFDN), and total choriocapillaris flow-deficit area (CCFDA). Correlations between imaging parameters and SER or AL were analyzed.

**Results:**

CT decreased significantly with increasing myopia severity at all measured macular locations. This reduction was driven mainly by thinning of Haller’s layer, whereas changes in SLCCT were smaller and more regionally restricted. Area-based analysis showed significant reductions in LA and TCA in the full-thickness choroid; sublayer analysis showed reduced LA and TCA in Haller’s layer and reduced SA and TCA in the Sattler’s layer–choriocapillaris complex. No significant between-group differences were observed in OCTA-derived perfusion or flow-deficit parameters. OCT-derived structural parameters were positively correlated with SER and negatively correlated with AL. Among OCTA-derived parameters, FCPD was correlated with SER, whereas CCPR, CCFD, and CCFDA showed weak correlations with AL.

**Conclusion:**

In school-aged children, increasing myopia severity was associated mainly with choroidal structural changes, including thinning of the total choroid, predominant involvement of Haller’s layer, and reductions in choroidal area-related parameters. OCTA-derived perfusion parameters showed no significant between-group differences, although limited associations with SER and AL were observed. These findings suggest that structural OCT-based metrics may better reflect early choroidal changes across refractive groups in children than OCTA-derived perfusion parameters.

## 1 Introduction

Myopia is an increasingly important public health problem, particularly in children and adolescents (1, 2). Earlier onset is associated with longer cumulative exposure to axial elongation and a higher lifetime risk of myopia-related ocular complications (3, 4). A better understanding of early ocular changes during childhood myopia progression is therefore of substantial clinical relevance (5, 6).

The choroid is considered an important tissue in the regulation of ocular growth (7). Previous studies have shown that thinner choroid is associated with longer axial length and more myopic refractive status, but the structural basis and functional significance of these changes remain incompletely understood (7, 8). Recent advances in enhanced depth imaging optical coherence tomography (EDI-OCT) and optical coherence tomography angiography (OCTA) now permit in vivo evaluation of choroidal thickness, vascular-stromal composition, and choriocapillaris perfusion (9, 10).

In pediatric populations, myopia-related choroidal changes have been reported in both structural optical coherence tomography (OCT) and OCTA studies (9–11). However, findings for perfusion-related parameters remain inconsistent, and most previous studies have focused on isolated metrics rather than integrated structural and perfusion profiling (7, 9, 12). In particular, studies combining full-thickness choroidal thickness, sublayer thickness, area-based structural parameters, and OCTA-derived perfusion indices in the same pediatric cohort remain limited.

Therefore, in the present retrospective analysis, we used EDI-OCT and OCTA to compare children with emmetropia, low myopia, and moderate myopia. We quantified full-thickness choroidal thickness (CT), Haller layer thickness (HLT), Sattler’s layer–choriocapillaris complex thickness (SLCCT), area-based structural parameters, and choriocapillaris perfusion-related indices, and analyzed their associations with spherical equivalent refraction (SER) and axial length (AL). The aim of this study was to determine whether childhood myopia is characterized predominantly by structural remodeling, perfusion changes, or both.

## 2 Materials and methods

### 2.1 Study population

This single-center retrospective cross-sectional study reviewed the clinical records and ophthalmic images of healthy children examined at the Ophthalmology Outpatient Department of the Affiliated Hospital of Inner Mongolia Medical University between October 2024 and October 2025. Clinical and imaging data from 150 potentially eligible participants were initially identified. The study adhered to the Declaration of Helsinki and was approved by the Ethics Committee of Inner Mongolia Medical University (Hohhot, China).

#### 2.1.1 Inclusion criteria

Participants were eligible if they met all of the following criteria: (1) age 8–14 years; (2) residence in urban areas of Hohhot City, China; (3) best-corrected visual acuity ≥ 0.8 and intraocular pressure (IOP) between 10 and 21 mmHg; (7) astigmatism ≤ 2.00 D; (8) interocular difference in spherical equivalent under non-accommodative conditions ≤ 2.00 D; and (13) no abnormalities on slit-lamp biomicroscopy or fundus examination.

#### 2.1.2 Exclusion criteria

Participants were excluded if they had systemic or ocular conditions that could affect ocular structure or blood flow, including hypertension and diabetes; a history of intraocular surgery, ocular trauma, or intravitreal injection; or previous use of orthokeratology lenses or multifocal soft contact lenses.

#### 2.1.3 Grouping

Participants were classified according to cycloplegic SER as follows: emmetropia (−0.50 D < SER ≤ + 0.75 D), low myopia (−3.00 D < SER ≤ −0.50 D), and moderate myopia (−6.00 D < SER ≤ −3.00 D). To avoid inter-eye correlation, only right-eye data were included in the analysis.

#### 2.1.4 Instruments and equipment

The instruments used in this study included a standard logarithmic visual acuity chart (GB11533-2011), an autorefractor (KR8800; Topcon, Tokyo, Japan), a slit-lamp biomicroscope (SL-D301; Topcon, Tokyo, Japan), a non-contact tonometer (TX-20; Canon, Tokyo, Japan), a direct ophthalmoscope (YZ11D; Suzhou Liuliu, Suzhou, China), an ocular biometer (IOLMaster 500; Carl Zeiss Meditec, Jena, Germany), and a spectral-domain optical coherence tomography system (Spectralis OCT; Heidelberg Engineering, Heidelberg, Germany).

### 2.2 Examination procedures

#### 2.2.1 Routine ophthalmic examination

For this retrospective analysis, demographic and clinical data, including age and sex, were extracted from the medical records. All included participants had undergone standardized ophthalmic examinations during routine clinical care, including uncorrected distance visual acuity testing, slit-lamp biomicroscopy, direct ophthalmoscopy, non-contact tonometry, ocular biometry, OCT, OCTA, and cycloplegic refraction. Uncorrected distance visual acuity was measured at 5 m using a standard logarithmic visual acuity chart. Slit-lamp biomicroscopy was used to evaluate the eyelids, conjunctiva, cornea, anterior chamber, iris, pupil, and lens. Direct ophthalmoscopy was performed to exclude retinal abnormalities. IOP was measured under natural pupil conditions using a non-contact tonometer. Three measurements were obtained for each eye, and the mean value was recorded.

#### 2.2.2 Ocular biometry and cycloplegic refraction

Before cycloplegia, AL was measured under natural pupil conditions using the IOLMaster 500. Measurements were repeated until the device quality criteria were met, and the mean value was used for analysis. Cycloplegic refraction was performed after three administrations of cyclopentolate hydrochloride eye drops at 5-min intervals. After adequate cycloplegia was achieved, objective refraction was performed and refined as needed. SER was calculated as spherical refractive error plus half of the cylindrical refractive error and was used as the primary grouping variable.

### 2.3 OCT and OCTA image acquisition

All OCT and OCTA scans included in this retrospective analysis had been acquired by trained ophthalmologists using the Spectralis OCT system according to a standardized protocol during routine clinical evaluation. Imaging was performed under natural pupil conditions before pharmacologic refraction.

For structural imaging, EDI-OCT was used to obtain a central horizontal foveal scan over a 6.0 × 6.0 mm area centered on the macula. Inverted image acquisition mode was used to improve visualization of the choroid–sclera junction (CSJ). The eye-tracking function was enabled during all scans to reduce motion artifacts and improve image quality.

For OCTA imaging, a 10° × 10° scan centered on the macula, corresponding to approximately 3.0 × 3.0 mm, was acquired. En face images of the full-thickness choroid and choriocapillaris were generated using the device software. The choriocapillaris slab was automatically defined by the device as extending from 9 μm above to 31 μm below Bruch’s membrane (BM). The full-thickness choroidal slab extended from the BM/RPE complex to the CSJ.

To reduce the potential influence of diurnal variation on choroidal measurements, according to the departmental imaging protocol, OCT and OCTA examinations were performed between 13:00 and 18:00.

### 2.4 Image quality control

All stored OCT and OCTA images were reviewed by a trained ophthalmologist before analysis. The quality of OCTA images was evaluated using the device-derived OCTA Quality metric, expressed in decibels (dB). Images with OCT Quality values below 20 dB and OCTA Quality values below 25 dB were excluded from the study (13, 14). Additionally, images exhibiting poor centration, motion artifacts, media opacity, insufficient signal strength, segmentation failures, or blurred boundaries that could compromise measurement reliability were excluded. The OCT and OCTA Quality values for the images that met the inclusion criteria were documented and compared across the three refractive groups.

### 2.5 Image annotation and quantitative analysis

Stored OCT and OCTA images were manually annotated using Labelme and subsequently analyzed using a custom Python script. The analysis workflow included image import, scale calibration, region-of-interest extraction based on annotated masks, grayscale normalization, lumen and stroma segmentation, and automated calculation of thickness, area-based, and perfusion-related parameters according to predefined formulas.

#### 2.5.1 OCT thickness measurement

For each eye, a horizontal EDI-OCT B-scan centered on the fovea was manually annotated using Labelme. Annotated landmarks included the BM/RPE complex, CSJ, the foveal center, and the boundary between Haller’s layer and the Sattler’s layer–choriocapillaris complex. The RPE-BM complex was defined as the outer border of the retinal pigment epithelium–Bruch’s membrane complex, the CSJ as the posterior choroidal boundary, and the foveal center as the point of maximal foveal depression on the horizontal scan.

The Haller–Sattler boundary was identified primarily according to vascular caliber and relative depth within the choroid, consistent with prior OCT-based analyses of choroidal sublayers in myopic eyes and children (15–17). Large hyporeflective vascular lumina adjacent to the CSJ were assigned to Haller’s layer, whereas smaller and medium-sized vascular lumina internal to these large vessels were assigned to the Sattler’s layer–choriocapillaris complex. Accordingly, the boundary was placed at the innermost margin of the large-vessel compartment. When the interface was indistinct, graders assigned the most anatomically plausible boundary based on overall vessel-size distribution and continuity of adjacent vascular profiles across the scan.

**FIGURE 1:**
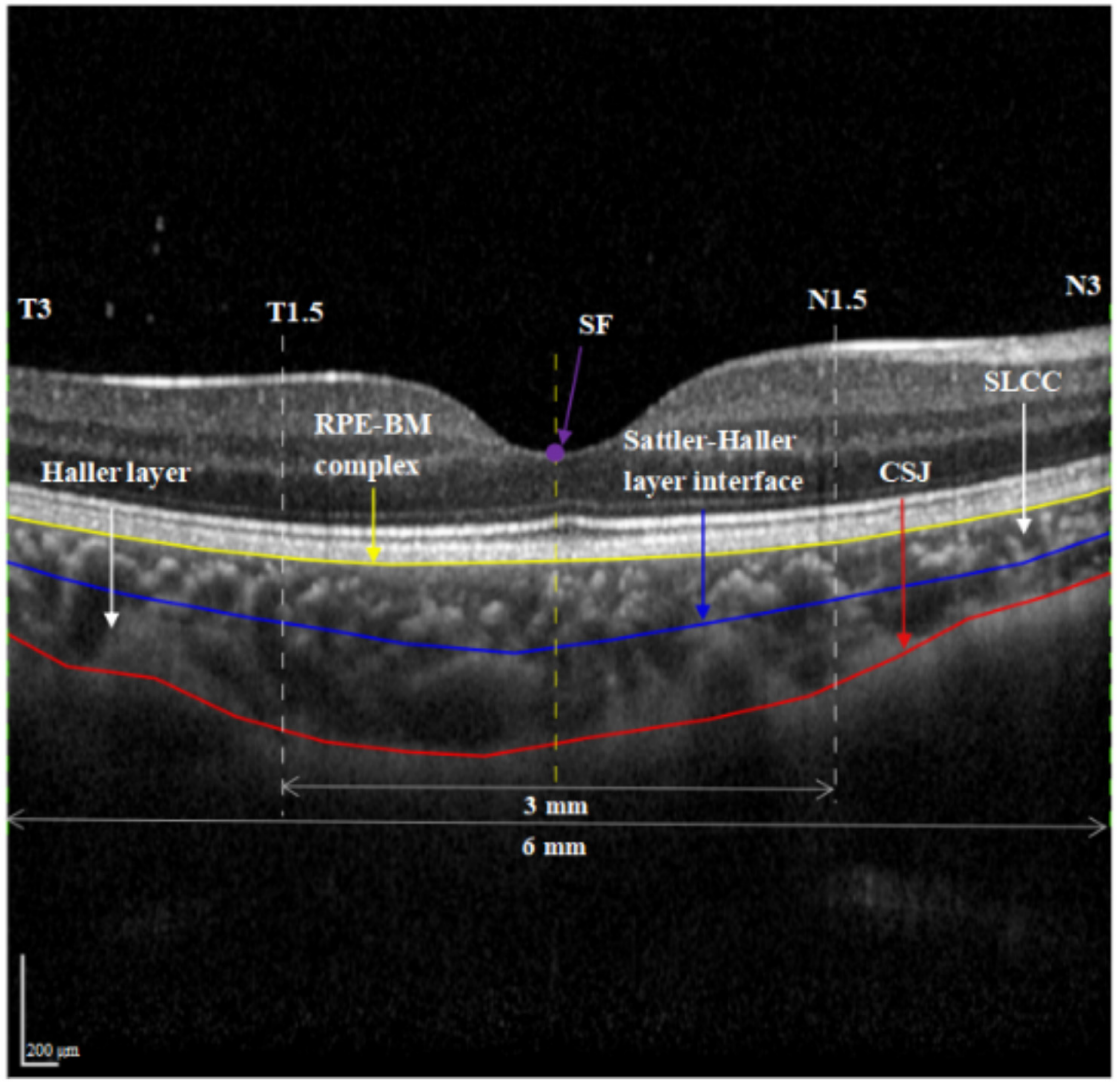
Representative manually annotated EDI-OCT B-scan used for choroidal layer segmentation and thickness measurement. The RPE-BM complex, Haller–Sattler interface, and CSJ were manually delineated. Thickness measurements were obtained at SF, N1.5, N3, T1.5, and T3.

CT was defined as the perpendicular distance from the RPE-BM complex to the CSJ and was measured at five predefined locations: subfoveal (SF), 1.5 mm nasal (N1.5), 3.0 mm nasal (N3), 1.5 mm temporal (T1.5), and 3.0 mm temporal (T3). Subfoveal choroidal thickness was defined as the CT directly beneath the foveal center. HLT was defined as the perpendicular distance from the Haller–Sattler boundary to the CSJ at each location. SLCCT was defined as the inner choroidal thickness and was calculated as CT minus HLT. Pixel-based measurements were converted to micrometers using image-specific scale information. Representative annotations are shown in Figure 1.

#### 2.5.2 Area-based structural measurements

For area-based structural analysis, EDI-OCT B-scan images were converted to grayscale and processed uniformly. Initially, images were denoised to enhance choroidal visibility, followed by binarization. Both raw and processed images were manually checked for clear anatomical boundaries. Using LabelMe software, the full-thickness choroidal region was outlined on each B-scan, with the upper boundary at the retinal pigment epithelium/Bruch’s membrane complex and the lower at the choroid–sclera interface. The ROI was limited to a 6 mm lateral extent, excluding areas beyond this.

For layer-specific analysis, the Haller–Sattler boundary was manually traced within the choroidal region of interest (ROI), marking the division between the inner medium-vessel zone and the outer large-vessel zone. The area from the upper choroidal border to this boundary was termed the Sattler layer–choriocapillaris complex, while the area from the boundary to the lower choroidal border was called the Haller layer. Grayscale intensities in each ROI were normalized and binarized. Low-reflectivity pixels were identified as luminal area (LA), and high-reflectivity pixels as stromal area (SA). Background pixels outside the ROI were ignored. The binary mask was reviewed for errors, which were corrected or discarded based on image quality standards. Total choroidal area (TCA) was calculated as the sum of LA and SA, with SA being the area remaining after LA segmentation. The choroidal vascularity index (CVI) was calculated as the ratio of LA to TCA and expressed as a percentage.

**FIGURE 2:**
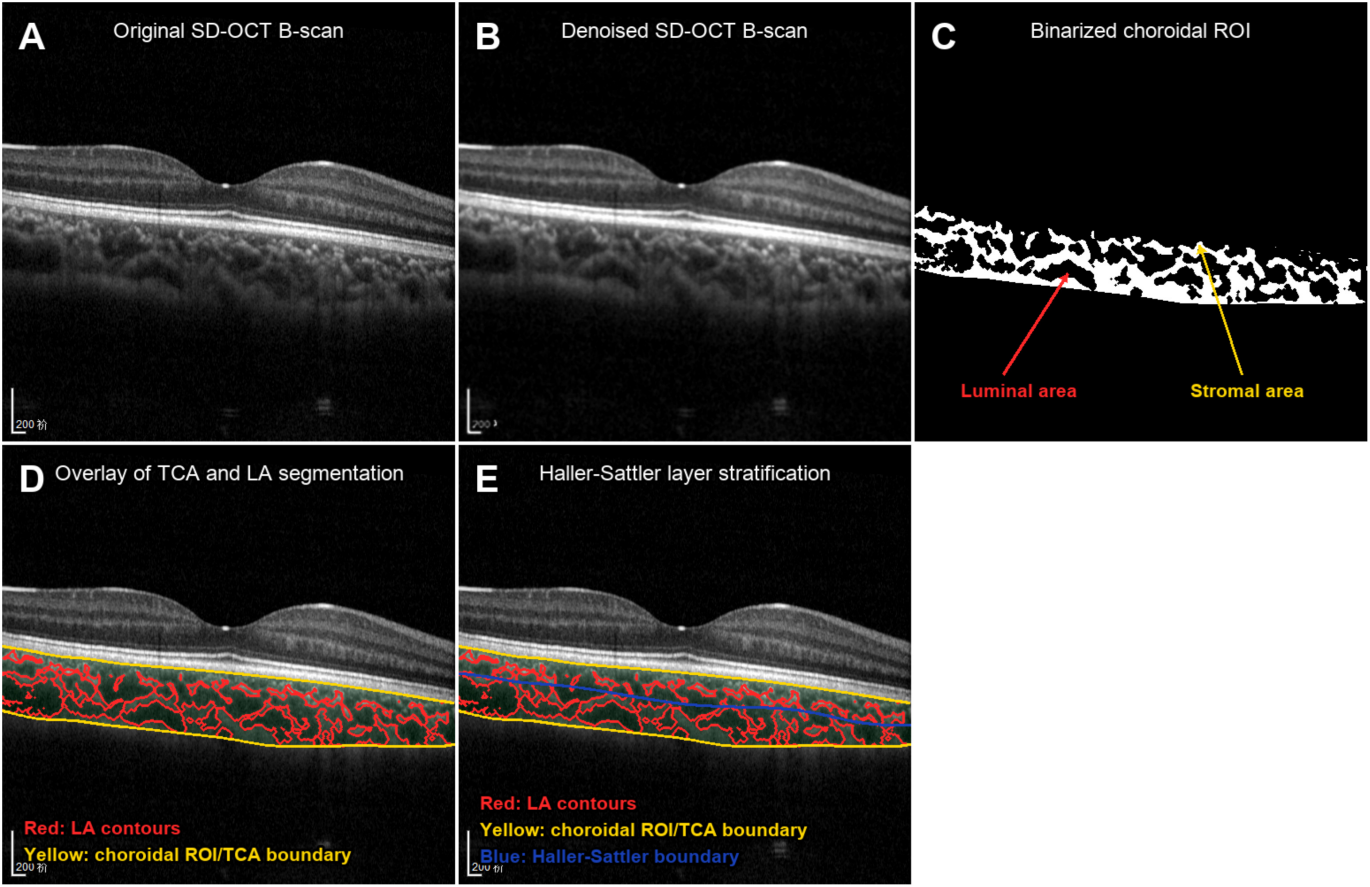
Workflow for total area and layered quantitative analysis of the chorioidal luminal and stromal areas on spectral-domain optical coherence tomography (SD-OCT) B-scans. **(A)** The original spectral-domain optical coherence tomography (SD-OCT) B-scan image. **(B)** The denoised SD-OCT B-scan image, utilized for subsequent binarization processes. **(C)** The binarized image focusing on the choroidal region of interest (ROI), where black pixels with low reflectance within the ROI are designated as the lacquer area (LA), and white pixels are identified as the stromal area (SA). Black background pixels located outside the ROI are omitted from quantitative analysis. **(D)** The segmentation overlay illustrating the boundary of the total choroidal area (TCA) alongside the LA contour. **(E)** The layered segmentation of the Haller/Sattler region based on the Haller–Sattler boundary, with the red contour delineating the LA boundary, the yellow contour marking the choroidal ROI/total choroidal area (ROI/TCA) boundary, and the blue line representing the Haller–Sattler boundary.

TCA, LA, SA, and CVI were computed separately for the total choroidal ROI, the Sattler’s layer–choriocapillaris complex subregion, and the Haller’s layer subregion. A representative workflow is shown in Figure 2.

#### 2.5.3 OCTA perfusion measurement

**FIGURE 3:**
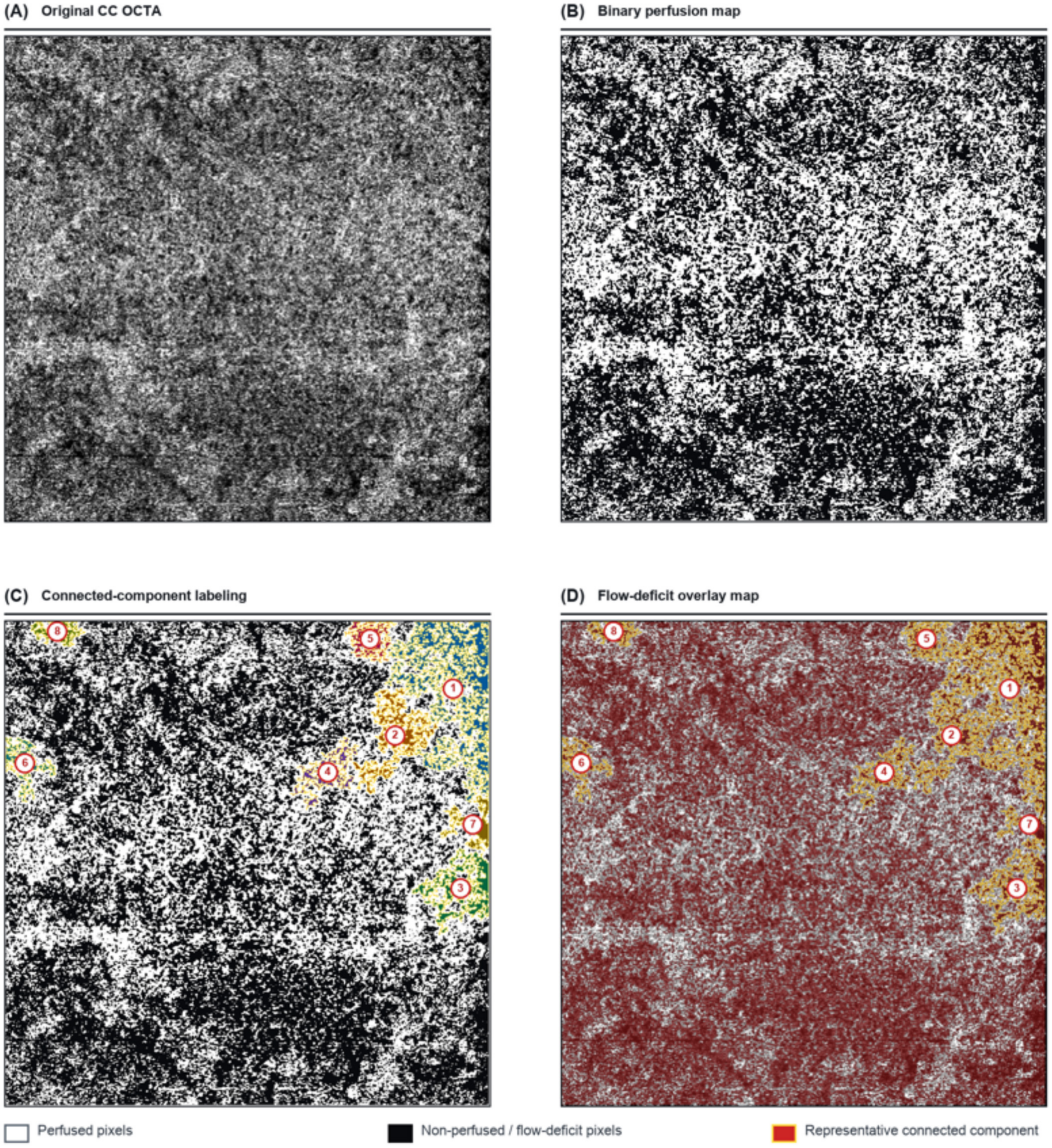
Workflow for binarization, labeling, and quantification of choriocapillaris OCTA flow-deficit regions. **(A)** Original en face OCTA image of the choriocapillaris slab. **(B)** Binary perfusion map generated after thresholding, in which white pixels indicate perfusion signal and black pixels indicate candidate flow-deficit pixels. **(C)** Labeling of contiguous flow-deficit regions on the binary mask; colored and numbered regions indicate representative discrete flow-deficit regions formed by adjacent flow-deficit pixels. **(D)** Flow-deficit overlay map showing the segmented flow-deficit pixels and representative flow-deficit regions superimposed on the original OCTA image. The choriocapillaris perfusion ratio (CCPR), choriocapillaris flow-deficit fraction (CCFD), choriocapillaris flow-deficit region number (CCFDN), and choriocapillaris flow-deficit average area (CCFDA) were calculated from the binary flow-deficit mask and region-labeling outputs but are not directly displayed in the schematic.

Full-thickness choroidal and choriocapillaris OCTA images were exported and analyzed using a Python script. Image scaling data was extracted for pixel-based area conversion. Before analysis, regions with significant artifacts or outside the image margins were excluded. Pixels outside the ROI were omitted from perfusion parameter calculations. For full-thickness choroidal images, full-choroid perfusion density (FCPD) was calculated as the ratio of pixels with detectable blood flow signals to the total analyzable pixels. The image processing workflow for choriocapillaris OCTA images is shown in Figure 3, with original images exported as grayscale (Figure 3A). These grayscale images were then binarized using a preset threshold method, which remained consistent across all included samples (Figure 3B). In the resulting binary maps, white pixels were classified as perfused pixels, while black pixels were classified as non-perfused or flow-deficient pixels. Low-intensity regions in the original OCTA images were not directly considered flow-deficient unless they persisted in the final binary flow-deficiency mask after thresholding and artifact exclusion. The total number of analyzable pixels within the choriocapillaris ROI served as the denominator for pixel-based choriocapillaris perfusion measurements.

The choriocapillaris perfusion ratio (CCPR) was defined as the proportion of perfused pixels within the analyzable CC region; conversely, the choriocapillaris flow deficiency percentage (CCFD) was defined as the proportion of flow-deficient pixels within the same region. Consequently, both CCPR and CCFD were derived from pixel classification in binary perfusion maps.

To further quantify the spatial distribution of choriocapillaris blood flow deficiencies, a connected component labeling algorithm was applied to the final binary flow-deficiency mask (Figure 3C). Pixels with 8-neighbor connectivity—defined as sharing an edge or corner—were assigned to the same labeled region to account for spatial continuity. To mitigate interference from isolated noise pixels and minute speckle-like artifacts, labeled regions with an area smaller than 600 pixels were excluded. The remaining labeled regions were defined as retained blood flow deficiency areas.

Choriocapillaris flow deficit number (CCFDN) was defined as the count of retained flow-deficit components after application of the 600-pixel area threshold within the analyzable choriocapillaris region. Choriocapillaris flow deficit average area (CCFDA) was calculated as the mean physical area of these retained components. For each component, pixel counts were converted to physical area (mm^2^) using the image-specific scale factor embedded in the exported OCTA dataset. The segmented flow-deficit regions were superimposed onto the native choriocapillaris OCTA image for visualization (Figure 3D). The color-coded and numbered regions depicted in the workflow diagram serve solely as illustrative examples of representative connected flow-deficit areas and are not employed as independent quantitative variables.

#### 2.5.4 Measurement procedure

Two trained ophthalmologists independently annotated all eligible images using Labelme, and quantitative parameter extraction was performed using custom Python scripts. The final value for each parameter was defined as the mean of the measurements obtained from the two independent annotations.

The annotation protocol and custom Python scripts used for image analysis are available from the corresponding author upon reasonable request.

### 2.6 Statistical analysis

Statistical analyses were performed using R version 4.4.3, and graphical representations were generated using GraphPad Prism 10.6. Continuous variables with normal distribution are presented as mean ± standard deviation. Comparisons among the three groups were performed using one-way analysis of variance (ANOVA) or Welch’s ANOVA, followed by Tukey’s honestly significant difference test or the Games–Howell test, as appropriate. Non-normally distributed variables are presented as median with interquartile range. These variables were compared using the Kruskal–Wallis test followed by Dunn’s test for pairwise comparisons. Categorical variables are presented as frequency and percentage and were compared using the chi-square test or Fisher’s exact test, as appropriate. Correlations between imaging parameters and clinical variables were analyzed using Pearson correlation when normality assumptions were met and Spearman rank correlation otherwise. All tests were two-sided, and *P* < 0.05 was considered statistically significant.

## 3 Results

### 3.1 Baseline demographic and ocular characteristics of study participants

Clinical records and imaging datasets from 150 potentially eligible children were reviewed. After image quality assessment, 10 datasets were excluded because of decentration, motion artifacts, insufficient signal strength, or segmentation failure. Intergroup comparisons of OCT and OCTA quality metrics were conducted among the emmetropic, low myopia, and moderate myopia groups to evaluate potential differences in image quality across the cohorts. The overall OCT image quality was 34.91 ± 4.55 dB, with no statistically significant differences observed among the emmetropic, low myopia, and moderate myopia groups (*F* = 1.396, *P* = 0.251). Similarly, the overall OCTA image quality was 34.89 ± 3.05 dB, with no statistically significant intergroup differences detected (*F* = 1.613, *P* = 0.203). These findings indicate that the image quality of the included scans was comparable across all groups (Supplementary Table 1). The final analysis included 140 children (140 right eyes): 30 in the emmetropia group, 70 in the low myopia group, and 40 in the moderate myopia group.

Females accounted for 47.86% of the cohort. Inter-group comparisons revealed no significant differences in sex distribution (χ^2^ = 0.491, *P* = 0.782), age (*F* = 1.692, *P* = 0.188), or IOP (*F* = 0.736, *P* = 0.481), indicating satisfactory baseline comparability. Mean ages were 9.40 ± 1.99 years, 9.67 ± 1.71 years, and 10.18 ± 1.91 years for the emmetropic, low-myopic, and moderate-myopic groups, respectively; the corresponding mean SER values were −0.11 ± 0.36 D, −1.59 ± 0.52 D, and −3.93 ± 0.88 D (*F* = 347.579, *P* < 0.001). AL were 23.47 ± 0.97 mm, 24.12 ± 0.73 mm, and 25.25 ± 0.87 mm, respectively (*F* = 42.878, *P* < 0.001). Mean IOPs were 16.17 ± 2.31 mmHg, 15.79 ± 1.92 mmHg, and 15.50 ± 2.75 mmHg in the three groups. These findings confirm the anticipated graded differences in refractive status and AL across groups, while other baseline characteristics remained broadly comparable. Detailed demographic and ocular baseline data are summarized in Table 1.

**TABLE 1:** Comparison of demographic and ocular baseline characteristics among the three study groups.

Characteristics	Emmetropia group (*n* = 30)	Low myopia group (*n* = 70)	Moderate myopia group (*n* = 40)	*F/χ* ^2^	*P*
Sex				0.491	0.782
Male	16 (53.33)	38 (54.29)	19 (47.5)		
Female	14 (46.67)	32 (45.71)	21 (52.5)		
Age (y)	9.4 ± 1.99	9.67 ± 1.71	10.18 ± 1.91	1.692	0.188
SER (D)	−0.11 ± 0.36	−1.59 ± 0.52	−3.93 ± 0.88	347.579	<0.001
AL (mm)	23.47 ± 0.97	24.12 ± 0.73	25.25 ± 0.87	42.878	<0.001
IOP (mmHg)	16.17 ± 2.31	15.79 ± 1.92	15.5 ± 2.75	0.736	0.481

Data are presented as n (%) or mean ± standard deviation, as appropriate. *P*-values were calculated using the chi-square test for sex and one-way analysis of variance for age, SER, AL, and IOP. SER, spherical equivalent refraction; AL, axial length; IOP, intraocular pressure.

### 3.2 Comparison of macular choroidal thickness among groups

Macular CT was compared among the emmetropia, low myopia, and moderate myopia groups at SF, N1.5, N3, T1.5, and T3. Significant between-group differences were observed at all five locations (all *P* < 0.05). Mean SF CT was 316.87 ± 55.23 μm in the emmetropia group, 277.86 ± 56.52 μm in the low myopia group, and 257.94 ± 53.24 μm in the moderate myopia group (*F* = 9.884, *P* < 0.001). The corresponding values at N1.5 were 262.53 ± 55.59 μm, 228.32 ± 60.07 μm, and 205.85 ± 52.30 μm (*F* = 8.490, *P* < 0.001); at N3, 182.04 ± 44.27 μm, 163.13 ± 43.97 μm, and 148.20 ± 43.48 μm (*F* = 5.094, *P* = 0.007); at T1.5, 328.06 ± 53.54 μm, 289.37 ± 57.15 μm, and 276.17 ± 45.81 μm (*F* = 8.602, *P* < 0.001); and at T3, 308.29 ± 51.21 μm, 276.99 ± 52.59 μm, and 263.07 ± 39.24 μm (*F* = 7.562, *P* < 0.001).

Post hoc analysis showed that CT at SF, N1.5, T1.5, and T3 was significantly lower in both myopia groups than in the emmetropia group (all *P* < 0.05). At N3, CT was significantly lower only in the moderate myopia group compared with the emmetropia group (*P* < 0.05). No significant difference was observed between the low and moderate myopia groups at any location. Detailed results are shown in Table 2.

**TABLE 2:** Comparison of choroidal thickness at different macular locations among the three study groups.

CT (μm)	Emmetropia group (*n* = 30)	Low myopia group (*n* = 70)	Moderate myopia group (*n* = 40)	*F*	*P*
SF	316.87 ± 55.23	277.86 ± 56.52^a^	257.94 ± 53.24^a^	9.884	<0.001
N1.5	262.53 ± 55.59	228.32 ± 60.07^a^	205.85 ± 52.3^a^	8.490	<0.001
N3	182.04 ± 44.27	163.13 ± 43.97	148.2 ± 43.48^a^	5.094	0.007
T1.5	328.06 ± 53.54	289.37 ± 57.15^a^	276.17 ± 45.81^a^	8.602	<0.001
T3	308.29 ± 51.21	276.99 ± 52.59^a^	263.07 ± 39.24^a^	7.562	<0.001

Data are presented as mean ± standard deviation. *P*-values were calculated using one-way analysis of variance, followed by *post hoc* pairwise comparisons. CT, choroidal thickness; SF, subfoveal; N1.5, nasal 1.5 mm; N3, nasal 3.0 mm; T1.5, temporal 1.5 mm; T3, temporal 3.0 mm. ^a^*P* < 0.05 compared with the emmetropia group.

### 3.3 Comparison of Haller layer thickness among groups

HLT was compared among the emmetropia, low myopia, and moderate myopia groups at SF, N1.5, N3, T1.5, and T3. Significant between-group differences were observed at all measured locations (all *P* < 0.05). Mean HLT at SF was 179.80 ± 46.54 μm in the emmetropia group, 152.96 ± 38.23 μm in the low myopia group, and 140.58 ± 33.13 μm in the moderate myopia group (*F* = 8.978, *P* < 0.001). The corresponding values at N1.5 were 143.44 ± 38.99 μm, 125.87 ± 39.09 μm, and 109.79 ± 28.35 μm (*F* = 7.388, *P* < 0.001); at N3, 101.43 ± 29.93 μm, 91.21 ± 29.62 μm, and 80.24 ± 26.83 μm (*F* = 4.677, *P* = 0.011); at T1.5, 190.86 ± 47.81 μm, 160.69 ± 41.34 μm, and 149.38 ± 29.74 μm (*F* = 9.695, *P* < 0.001); and at T3, 178.32 ± 48.70 μm, 156.61 ± 41.14 μm, and 138.27 ± 30.45 μm (*F* = 8.507, *P* < 0.001).

Post hoc analysis showed that HLT was significantly lower in the moderate myopia group than in the emmetropia group at all locations (all *P* < 0.05). In the low myopia group, HLT was also significantly lower at SF, T1.5, and T3 (all *P* < 0.05), but not at N1.5 or N3. No significant between-group difference was observed between the low and moderate myopia groups at any location. Detailed results are shown in Table 3.

**TABLE 3:** Comparison of Haller layer thickness at different macular locations among the three study groups.

HLT (μm)	Emmetropia group (*n* = 30)	Low myopia group (*n* = 70)	Moderate myopia group (*n* = 40)	*F*	*P*
SF	179.8 ± 46.54	152.96 ± 38.23^a^	140.58 ± 33.13^a^	8.978	< 0.001
N1.5	143.44 ± 38.99	125.87 ± 39.09	109.79 ± 28.35^a^	7.388	< 0.001
N3	101.43 ± 29.93	91.21 ± 29.62	80.24 ± 26.83^a^	4.677	0.011
T1.5	190.86 ± 47.81	160.69 ± 41.34^a^	149.38 ± 29.74^a^	9.695	< 0.001
T3	178.32 ± 48.7	156.61 ± 41.14^a^	138.27 ± 30.45^a^	8.507	< 0.001

Data are presented as mean ± standard deviation. *P*-values were calculated using one-way analysis of variance, followed by *post hoc* pairwise comparisons. HLT, Haller layer thickness; SF, subfoveal; N1.5, nasal 1.5 mm; N3, nasal 3.0 mm; T1.5, temporal 1.5 mm; T3, temporal 3.0 mm. ^a^*P* < 0.05 compared with the emmetropia group.

### 3.4 Comparison of SLCCT among groups

SLCCT was compared among the emmetropia, low myopia, and moderate myopia groups at 5 macular locations: SF, N1.5, N3, T1.5, and T3. Significant intergroup differences were observed at SF, N1.5, and N3 (all *P* < 0.05), whereas no significant differences were detected at T1.5 (*F* = 1.979, *P* = 0.142) or T3 (*F* = 2.513, *P* = 0.085).

Post hoc analyses showed that SLCCT was significantly lower in both myopia groups than in the emmetropia group at SF and N1.5 (all *P* < 0.05). At N3, SLCCT was significantly lower in the moderate myopia group than in the emmetropia group (*P* < 0.05), whereas the difference between the emmetropia and low myopia groups was not significant. No significant differences were observed between the low and moderate myopia groups at any location. Overall, SLCCT showed a decreasing trend with increasing myopia severity, particularly at the subfoveal and nasal locations. Detailed results are presented in Table 4.

**TABLE 4:** Comparison of SLCCT at different macular locations among the three study groups.

SLCCT (μm)	Emmetropia group (*n* = 30)	Low myopia group (*n* = 70)	Moderate myopia group (*n* = 40)	*F*	*P*
SF	137.07 ± 19.94	124.89 ± 24.41^a^	117.36 ± 23.83^a^	6.123	0.003
N1.5	119.09 ± 22.15	102.45 ± 26.66^a^	96.07 ± 26.36^a^	7.213	0.001
N3	80.60 ± 21.36	71.92 ± 19.80	67.96 ± 19.62^a^	3.487	0.033
T1.5	137.20 ± 21.03	128.67 ± 24.20	126.78 ± 22.14	1.979	0.142
T3	129.97 ± 18.18	120.39 ± 21.31	124.80 ± 18.62	2.513	0.085

Data are presented as mean ± standard deviation. *P*-values were calculated using one-way analysis of variance, followed by *post hoc* pairwise comparisons. SLCCT, Sattler’s layer–choriocapillaris complex thickness; SF, subfoveal; N1.5, nasal 1.5 mm; N3, nasal 3.0 mm; T1.5, temporal 1.5 mm; T3, temporal 3.0 mm. ^a^*P* < 0.05 compared with the emmetropia group.

### 3.5 Comparison of area-based structural parameters among groups

At the full-thickness choroidal level, significant between-group differences were observed for SA, LA, TCA, and CVI (all *P* ≤ 0.028). SA was significantly lower in both myopia groups than in the emmetropia group (0.64 ± 0.11 mm^2^, 0.57 ± 0.10 mm^2^, and 0.57 ± 0.09 mm^2^; *F* = 6.013, *P* = 0.003). LA and TCA also decreased with increasing myopia severity (LA: 1.13 ± 0.25 mm^2^, 0.99 ± 0.27 mm^2^, and 0.88 ± 0.23 mm^2^; *F* = 7.790, *P* < 0.001; TCA: 1.76 ± 0.29 mm^2^, 1.55 ± 0.31 mm^2^, and 1.45 ± 0.27 mm^2^; *F* = 9.795, *P* < 0.001). CVI also differed significantly among groups, with the lowest value observed in the moderate myopia group (63.41 ± 5.77%, 62.84 ± 6.38%, and 59.88 ± 6.41%; *F* = 3.660, *P* = 0.028).

In Haller’s layer, significant between-group differences were observed for LA and TCA (LA: 0.87 ± 0.26 mm^2^, 0.73 ± 0.23 mm^2^, and 0.65 ± 0.19 mm^2^; *F* = 8.460, *P* < 0.001; TCA: 1.01 ± 0.24 mm^2^, 0.87 ± 0.21 mm^2^, and 0.79 ± 0.16 mm^2^; *F* = 9.858, *P* < 0.001), whereas no significant differences were observed for SA or CVI (SA: 0.14 ± 0.06 mm^2^, 0.14 ± 0.06 mm^2^, and 0.15 ± 0.06 mm^2^; *F* = 0.260, *P* = 0.771; CVI: 85.53 ± 7.75%, 82.51 ± 9.41%, and 80.12 ± 10.19%; *F* = 2.883, *P* = 0.059).

In the Sattler’s layer–choriocapillaris complex, significant between-group differences were observed for SA and TCA (SA: 0.51 ± 0.09 mm^2^, 0.43 ± 0.10 mm^2^, and 0.43 ± 0.09 mm^2^; *F* = 7.617, *P* < 0.001; TCA: 0.78 ± 0.10 mm^2^, 0.71 ± 0.13 mm^2^, and 0.68 ± 0.12 mm^2^; *F* = 5.880, *P* = 0.004), whereas no significant differences were found for LA or CVI (LA: 0.27 ± 0.08 mm^2^, 0.28 ± 0.09 mm^2^, and 0.25 ± 0.06 mm^2^; *F* = 1.453, *P* = 0.237; CVI: 34.53 ± 8.22%, 38.89 ± 9.27%, and 36.61 ± 6.67%; *F* = 3.038, *P* = 0.051). Detailed results are shown in Table 5.

**TABLE 5:** Comparison of LA, SA, TCA, and CVI in the full-thickness choroid and its sublayers among groups.

Layer and parameter	Emmetropia group (*n* = 30)	Low myopia group (*n* = 70)	Moderate myopia group (*n* = 40)	*F*	*P*
Total choroid					
SA	0.64 ± 0.11	0.57 ± 0.10^a^	0.57 ± 0.09^a^	6.013	0.003
LA	1.13 ± 0.25	0.99 ± 0.27^a^	0.88 ± 0.23^a^	7.790	<0.001
TCA	1.76 ± 0.29	1.55 ± 0.31^a^	1.45 ± 0.27^a^	9.795	<0.001
CVI	63.41 ± 5.77	62.84 ± 6.38	59.88 ± 6.41^b^	3.660	0.028
Haller_layer					
SA	0.14 ± 0.06	0.14 ± 0.06	0.15 ± 0.06	0.260	0.771
LA	0.87 ± 0.26	0.73 ± 0.23^a^	0.65 ± 0.19^a^	8.460	<0.001
TCA	1.01 ± 0.24	0.87 ± 0.21^a^	0.79 ± 0.16^a^	9.858	<0.001
CVI	85.53 ± 7.75	82.51 ± 9.41	80.12 ± 10.19^a^	2.883	0.059
Sattler’s layer–choriocapillaris complex					
SA	0.51 ± 0.09	0.43 ± 0.10^a^	0.43 ± 0.09^a^	7.617	<0.001
LA	0.27 ± 0.08	0.28 ± 0.09	0.25 ± 0.06	1.453	0.237
TCA	0.78 ± 0.1	0.71 ± 0.13^a^	0.68 ± 0.12^a^	5.88	0.004
CVI	34.53 ± 8.22	38.89 ± 9.27^a^	36.61 ± 6.67	3.038	0.051

LA, luminal area; SA, stromal area; TCA, total choroidal area; CVI, choroidal vascularity index (LA/TCA × 100%). LA, SA, and TCA are expressed in mm^2^, and CVI is expressed as %. Data are presented as mean ± standard deviation. *P*-values were calculated using one-way analysis of variance, followed by *post hoc* pairwise comparisons. ^a^*P* < 0.05 compared with the emmetropia group; ^b^*P* < 0.05 compared with the low myopia group.

### 3.6 Comparison of OCTA perfusion parameters among groups

No significant between-group differences were observed in any OCTA-derived full choroidal or choriocapillaris perfusion parameter (all *P* > 0.05). FCPD values were 32.02 ± 10.09%, 33.90 ± 6.24%, and 35.77 ± 5.92% in the emmetropia, low myopia, and moderate myopia groups, respectively (*F* = 2.388, *P* = 0.096). CCPR values were 34.89 ± 9.26%, 36.08 ± 6.34%, and 36.78 ± 5.39% (*F* = 0.665, *P* = 0.516), and CCFD values were 65.11 ± 9.26%, 63.92 ± 6.34%, and 63.22 ± 5.39% (*F* = 0.665, *P* = 0.516). CCFDN values were 2091.80 ± 887.40, 2203.61 ± 750.14, and 2219.18 ± 684.40 (*F* = 0.284, *P* = 0.753), and CCFDA values were 5.80 ± 0.82 mm^2^, 5.70 ± 0.57 mm^2^, and 5.64 ± 0.49 mm^2^ (*F* = 0.628, *P* = 0.535). Detailed results are shown in Table 6.

**TABLE 6:** Comparison of full choroidal and choriocapillaris perfusion parameters among the emmetropia, low myopia, and moderate myopia groups.

OCTA parameter	Emmetropia group (*n* = 30)	Low myopia group (*n* = 70)	Moderate myopia group (*n* = 40)	*F*	*P*
FCPD (%)	32.02 ± 10.09	33.90 ± 6.24	35.77 ± 5.92	2.388	0.096
CCPR(%)	34.89 ± 9.26	36.08 ± 6.34	36.78 ± 5.39	0.665	0.516
CCFD (%)	65.11 ± 9.26	63.92 ± 6.34	63.22 ± 5.39	0.665	0.516
CCFDN (n)	2091.8 ± 887.4	2203.61 ± 750.14	2219.18 ± 684.40	0.284	0.753
CCFDA (mm^2^)	5.80 ± 0.82	5.70 ± 0.57	5.64 ± 0.49	0.628	0.535

Data are presented as mean ± standard deviation. *P*-values were calculated using one-way analysis of variance. FCPD, full-layer choroidal perfusion density; CCPR, choriocapillaris perfusion ratio; CCFD, choriocapillaris flow-deficit percentage; CCFDN, choriocapillaris flow-deficit number; CCFDA, choriocapillaris flow-deficit area.

### 3.7 Correlation analysis

#### 3.7.1 Correlation of OCT-derived structural parameters with SER and AL

**FIGURE 4:**
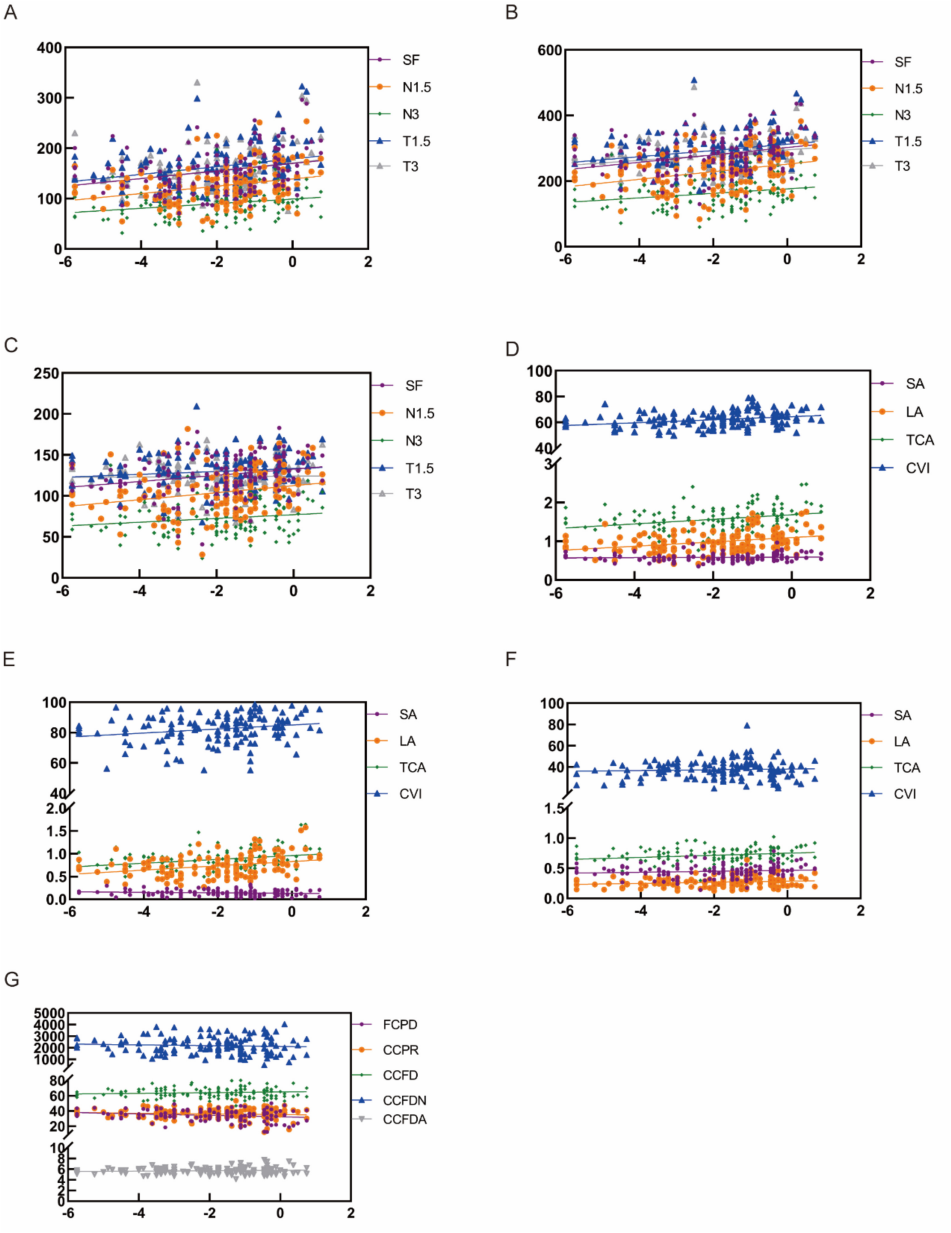
Correlation between OCT- and OCTA-derived parameters and SER. Scatter plots with regression lines illustrate the relationships between SER and parameters derived from OCT and OCTA. Panels **(A–C)** depict thickness metrics for the total choroid, Haller’s layer, and Sattler’s layer–choriocapillaris complex, respectively, while panels **(D–F)** present the corresponding area metrics. Panel **(G)** displays full-thickness choroidal and choriocapillaris perfusion parameters obtained via OCTA. Abbreviations: SF, subfoveal; N1.5, 1.5 mm nasal; N3, 3.0 mm nasal; T1.5, 1.5 mm temporal; T3, 3.0 mm temporal; SA, stromal area; LA, luminal area; TCA, total choroidal area; CVI, choroidal vascularity index; FCPD, full-thickness choroidal perfusion density; CCPR, choriocapillaris perfusion ratio; CCFD, choriocapillaris flow deficit percentage; CCFDN, choriocapillaris flow deficit number; CCFDA, choriocapillaris flow deficit area.

**FIGURE 5:**
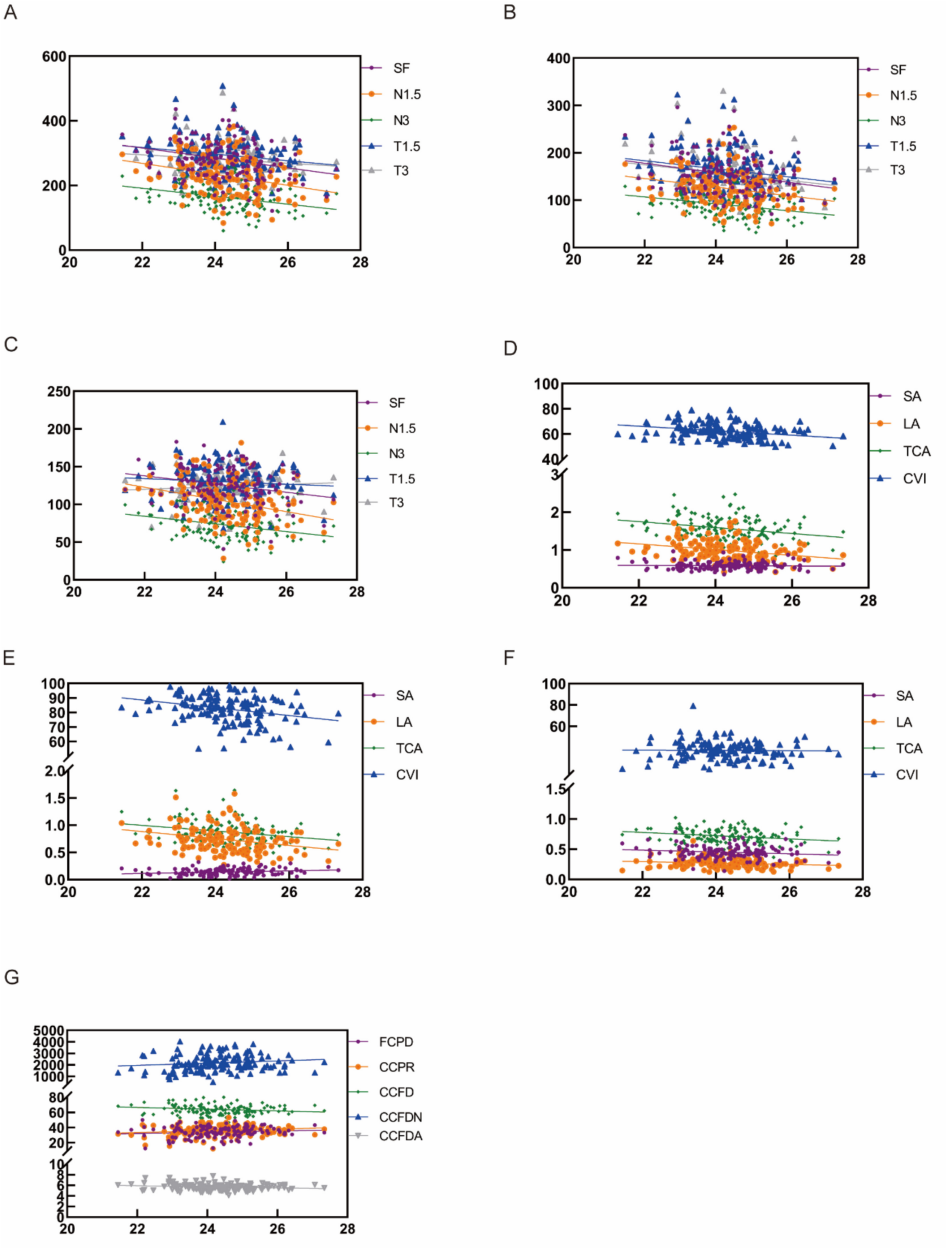
Correlations between AL and OCT and OCTA parameters. Scatter plots with regression fit lines illustrate the relationships between AL and OCT/OCTA parameters. **(A–C)** The thickness parameters of the total choroid, Haller’s layer, and Sattler’s layer–choriocapillaris complex, respectively. **(D–F)** The corresponding area parameters of these three layers. **(G)** The perfusion parameters of the full-thickness choroid and choriocapillaris obtained via OCTA.

As shown in Figures 4, 5, OCT-derived structural parameters were generally positively correlated with SER and negatively correlated with AL. Full-thickness choroidal thickness was positively correlated with SER at all five macular locations (SF: *r* = 0.292, *P* < 0.001; N1.5: *r* = 0.293, *P* < 0.001; N3: *r* = 0.232, *P* = 0.0058; T1.5: *r* = 0.256, *P* = 0.0022; T3: *r* = 0.238, *P* = 0.0047) and negatively correlated with AL at SF, N1.5, N3, and T1.5 (SF: *r* = −0.274, *P* = 0.0011; N1.5: *r* = −0.298, *P* < 0.001; N3: *r* = −0.285, *P* < 0.001; T1.5: *r* = −0.191, *P* = 0.0236), whereas the correlation at T3 was not significant (*r* = −0.134, *P* = 0.114).

HLT showed a similar pattern, with positive correlations with SER at all five locations (SF: *r* = 0.278, *P* < 0.001; N1.5: *r* = 0.291, *P* < 0.001; N3: *r* = 0.235, *P* = 0.0052; T1.5: *r* = 0.276, *P* < 0.001; T3: *r* = 0.278, *P* < 0.001) and negative correlations with AL at all locations (SF: *r* = −0.254, *P* = 0.0025; N1.5: *r* = −0.248, *P* = 0.0032; N3: *r* = −0.255, *P* = 0.0024; T1.5: *r* = −0.208, *P* = 0.0135; T3: *r* = −0.202, *P* = 0.0168). By contrast, SLCCT showed weaker and more regionally limited correlations. Positive correlations with SER were observed only at SF, N1.5, and N3 (SF: *r* = 0.238, *P* = 0.0047; N1.5: *r* = 0.243, *P* = 0.0038; N3: *r* = 0.172, *P* = 0.0423), and negative correlations with AL were also limited to SF, N1.5, and N3 (SF: *r* = −0.235, *P* = 0.0051; N1.5: *r* = −0.316, *P* < 0.001; N3: *r* = −0.260, *P* = 0.0019).

Area-based OCT parameters showed a similar overall pattern. In the full-thickness choroid, LA, TCA, and CVI were positively correlated with SER (LA: *r* = 0.317, *P* < 0.001; TCA: *r* = 0.289, *P* < 0.001; CVI: *r* = 0.285, *P* < 0.001) and negatively correlated with AL (LA: *r* = −0.294, *P* < 0.001; TCA: *r* = −0.265, *P* = 0.0016; CVI: *r* = −0.296, *P* < 0.001), whereas SA was not significantly correlated with SER or AL. In Haller’s layer, LA, TCA, and CVI were also positively correlated with SER (LA: *r* = 0.300, *P* < 0.001; TCA: *r* = 0.299, *P* < 0.001; CVI: *r* = 0.212, *P* = 0.0119) and negatively correlated with AL (LA: *r* = −0.282, *P* < 0.001; TCA: *r* = −0.257, *P* = 0.0022; CVI: *r* = −0.297, *P* < 0.001). Haller’s layer SA showed no significant correlation with SER and only a weak positive correlation with AL (*r* = 0.202, *P* = 0.0166). In the Sattler’s layer–choriocapillaris complex, both LA and TCA were positively correlated with SER (LA: *r* = 0.176, *P* = 0.0380; TCA: *r* = 0.211, *P* = 0.0123), whereas only TCA was negatively correlated with AL (*r* = −0.225, *P* = 0.0074).

#### 3.7.2 Correlation between OCTA-derived perfusion parameters and SER and AL

Compared with OCT-derived structural parameters, OCTA-derived perfusion biomarkers showed weaker associations with SER and AL (Figures 4, 5). Among all OCTA parameters, only FCPD was significantly negatively correlated with SER (*r* = −0.204, *P* = 0.0154), while CCPR, CCFD, CCFDN, and CCFDA showed no significant associations with SER (all *P* > 0.05).

Regarding AL, CCPR showed a weak positive correlation (*r* = 0.177, *P* = 0.0364), and CCFD and CCFDA showed weak negative correlations (*r* = −0.177, *P* = 0.0364; *r* = −0.174, *P* = 0.0397, respectively). Neither FCPD nor CCFDN showed significant correlations with AL (*P* = 0.153 and 0.114, respectively). Collectively, OCTA-derived perfusion parameters demonstrated limited associations with refractive and axial indices, with correlation strengths lower than those of OCT-derived structural biomarkers.

#### 3.7.3 Correlations among OCT-derived structural parameters and between OCT- and OCTA-derived parameters

**FIGURE 6:**
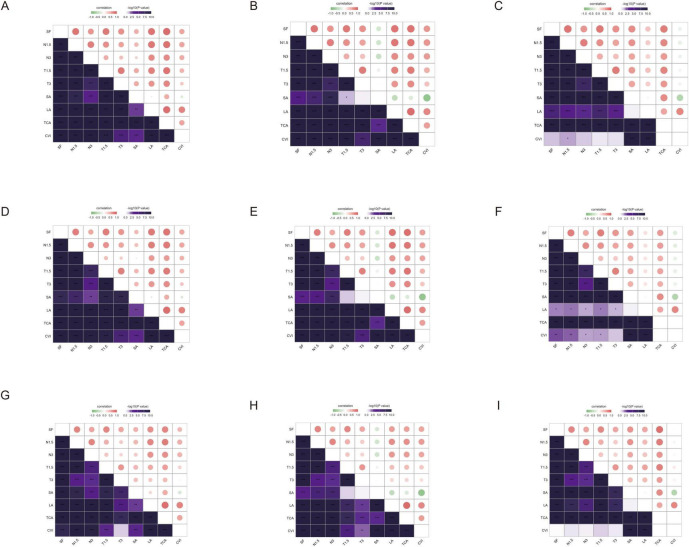
Heatmap of correlations among OCT-derived structural parameters. The heatmap summarizes pairwise correlations between thickness parameters at five macular locations and area structural parameters of the total choroid and its sublayers. **(A–C)** Correlations of full-thickness choroidal thickness with area parameters of the full-thickness choroid, Haller’s layer, and Sattler’s layer–choriocapillaris complex, respectively. **(D–F)** associations of Haller’s layer thickness with area parameters of the full-thickness choroid, Haller’s layer, and Sattler’s layer–choriocapillaris complex, respectively. **(G–I)** Associations of Sattler–choriocapillaris layer thickness with area parameters of the aforementioned layers. In the upper triangle, dot color indicates correlation direction, and dot size reflects the absolute value of the correlation coefficient; in the lower triangle, color intensity represents statistical significance as - log10 (adjusted *P*-value). Asterisks indicate statistically significant correlations. SF, subfoveal; N1.5, nasal 1.5 mm; N3, nasal 3.0 mm; T1.5, temporal 1.5 mm; T3, temporal 3.0 mm; SA, stromal area; LA, luminal area; TCA, total choroidal area; CVI, choroidal vascularity index. Asterisks: *P < 0.05, **P < 0.01, ***P < 0.001.

The OCT heatmap in Figure 6 demonstrates high internal correlations among OCT-derived structural parameters. At the overall choroidal level, thickness at each macular location was strongly correlated with LA, TCA, and CVI of the full-thickness choroid. Among these, thickness at SF showed the strongest correlations with full-thickness choroidal TCA (*r* = 0.967, *P* < 0.001) and LA (*r* = 0.918, *P* < 0.001); thickness at N1.5 was also highly correlated with full-thickness choroidal TCA (*r* = 0.936, *P* < 0.001) and LA (*r* = 0.902, *P* < 0.001). HLT showed similar patterns, with significant correlations with LA and TCA of this layer, particularly prominent at SF (*r* = 0.931 and 0.950; both *P* < 0.001) and N1.5 (*r* = 0.901 and 0.906; both *P* < 0.001). SLCCT also showed strong positive correlations with SA and TCA in the same region, especially significant at SF (*r* = 0.710 and 0.946; both *P* < 0.001) and N1.5 (*r* = 0.717 and 0.866; both *P* < 0.001).

**FIGURE 7:**
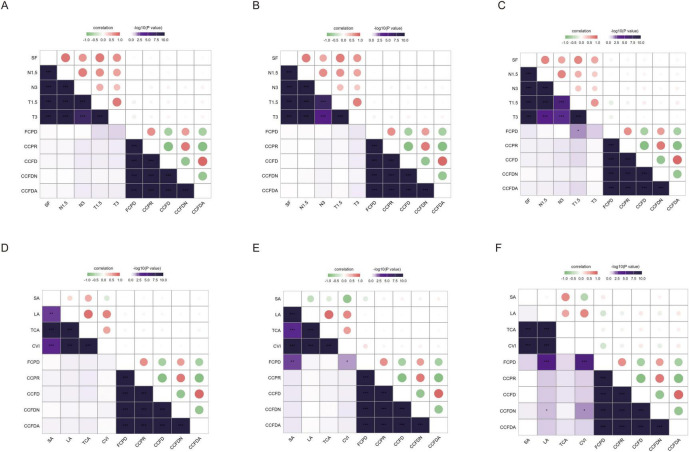
Heatmap of correlations between OCT-derived structural parameters and OCTA-derived perfusion biomarkers. The heatmap summarizes pairwise correlations between OCT structural parameters and OCTA perfusion biomarkers. **(A–C)** Correlations of thickness parameters of the full-thickness choroid, Haller’s layer, and Sattler’s layer–choriocapillaris complex with FCPD, CCPR, CCFD, CCFDN, and CCFDA, respectively. **(D–F)** Corresponding correlations of area parameters of the aforementioned layers with OCTA perfusion biomarkers. In the upper triangle, dot color indicates correlation direction, and dot size reflects the absolute value of the correlation coefficient; in the lower triangle, color intensity represents statistical significance as - log10 (adjusted *P*-value). Asterisks indicate statistically significant correlations. SF, subfoveal; N1.5, nasal 1.5 mm; N3, nasal 3.0 mm; T1.5, temporal 1.5 mm; T3, temporal 3.0 mm; SA, stromal area; LA, luminal area; TCA, total choroidal area; CVI, choroidal vascularity index; FCPD, full-thickness choroidal perfusion density; CCPR, choriocapillaris perfusion ratio; CCFD, choriocapillaris flow deficit percentage; CCFDN, choriocapillaris flow deficit number; CCFDA, choriocapillaris flow deficit area. Asterisks indicate statistically significant correlations.

Cross-layer correlations were also evident. Full-thickness choroidal thickness was strongly correlated with Haller’s layer LA and TCA (SF: *r* = 0.913 and 0.919; both *P* < 0.001), as well as with SLCCT SA and TCA (SF: *r* = 0.752 and 0.852; both *P* < 0.001). HLT was significantly positively correlated with full-thickness choroidal LA and TCA (SF: *r* = 0.882 and 0.913; both *P* < 0.001), and with Sattler’s layer -choriocapillaris complex SA and TCA (SF: *r* = 0.659 and 0.663; both *P* < 0.001). SLCCT was also significantly positively correlated with area parameters of the full-thickness choroid and Haller’s layer, particularly with full-thickness choroidal TCA (SF: *r* = 0.803, *P* < 0.001) and Haller’s layer LA (*r* = 0.640, *P* < 0.001). In contrast, correlations between OCT structural parameters and OCTA perfusion biomarkers were generally weak (Figure 7). Total choroidal thickness showed no significant correlations with FCPD, CCPR, CCFD, CCFDN, or CCFDA in any macular region (all *P* > 0.05). Similarly, HLT was not significantly associated with any OCTA biomarkers (all *P* > 0.05). In the Sattler’s layer-choriocapillaris complex, only T1.5 thickness showed a weak negative correlation with FCPD (*r* = −0.199, *P* = 0.0187), while all other thickness-OCTA associations were not statistically significant.

Area-based OCT-OCTA correlations were also limited. SA, LA, TCA, and CVI of the full-thickness choroid were not significantly correlated with any OCTA perfusion biomarkers (all *P* > 0.05). In Haller’s layer, SA showed a weak negative correlation with FCPD (*r* = −0.253, *P* = 0.0025), while CVI showed a weak positive correlation with FCPD (*r* = 0.191, *P* = 0.0239); no other Haller’s layer area parameters were significantly correlated with OCTA biomarkers. In the Sattler’s layer-choriocapillaris complex, both LA and CVI were negatively correlated with FCPD (LA: *r* = −0.353, *P* < 0.001; CVI: *r* = −0.373, *P* < 0.001), and both were also negatively correlated with CCFDN (LA: *r* = −0.167, *P* = 0.0484; CVI: *r* = −0.198, *P* = 0.0188). No other correlations were statistically significant.

Overall, OCT structural parameters demonstrated high internal consistency, whereas correlations between OCT structural biomarkers and OCTA perfusion parameters were relatively weak and selective.

## 4 Discussion

In this retrospective cross-sectional study of school-aged children, choroidal structural parameters differed across refractive groups, whereas OCTA-derived perfusion parameters did not. Choroidal thinning became more pronounced with increasing myopia severity and was mainly attributable to thinning of Haller’s layer. Area-based analysis further showed reductions in luminal and total choroidal area. By contrast, OCTA-derived perfusion and flow-deficit parameters showed no significant between-group differences and only weak associations with SER or AL. These findings suggest that, in childhood myopia up to the moderate stage, choroidal remodeling is characterized predominantly by structural rather than measurable perfusion changes.

The observed reduction in choroidal thickness is consistent with the current understanding of the choroid as a dynamic tissue involved in ocular growth regulation (6, 7, 18). Previous human studies have shown that thinner choroid is associated with longer axial length and more myopic refractive status (5, 6, 8, 19). In the present study, choroidal thinning was observed across all measured macular locations, with particularly clear reductions at the subfoveal and nasal sites. This spatial pattern is compatible with previously described topographic variation of the pediatric choroid and supports the presence of regionally distributed remodeling during myopia progression (12, 20, 21).

A key finding of this study is that myopia-related choroidal thinning was driven mainly by reduction in Haller layer thickness. This pattern suggests that early childhood myopia may preferentially affect the large-vessel choroidal compartment. This interpretation is biologically plausible because the outer choroidal vasculature may be more sensitive to axial elongation and biomechanical stretching (22–24). However, this observation should be interpreted cautiously. Previous studies have reported heterogeneous findings for choroidal sublayers, and differences in age, refractive range, imaging modality, segmentation strategy, and analytical methods may account for part of the variability (7, 23, 25–27). Our results therefore support the view that, within the refractive range studied here, Haller layer remodeling is a prominent feature of early myopia, rather than establishing that it is the only sublayer involved.

The area-based findings further support the presence of structural remodeling. In the full-thickness choroid, SA, LA, and TCA were reduced in the myopia groups, whereas CVI showed a smaller magnitude of change. At the sublayer level, Haller’s layer was characterized mainly by reductions in LA and TCA, whereas the Sattler’s layer–choriocapillaris complex showed reductions in SA and TCA. Taken together, these findings suggest that early myopic remodeling involves contraction of both total tissue area and vascular luminal area, with nonuniform distribution across choroidal sublayers. This pattern is consistent with prior studies showing that absolute structural parameters, including choroidal thickness, luminal area, total choroidal area, and vascularity-related indices, are altered in pediatric or young myopic eyes (6, 25, 26, 28, 29).

No significant between-group differences were observed in OCTA-derived perfusion parameters. This negative finding is still informative. Previous pediatric OCTA studies have reported inconsistent results, with some studies showing reduced choriocapillaris perfusion or increased flow-deficit burden in myopic children, whereas others have found minimal or no clear alterations in physiological myopia (5, 9, 30–32). In the present cohort, the absence of significant differences in FCPD, CCPR, CCFD, CCFDN, and CCFDA suggests that measurable choriocapillaris perfusion abnormalities may be limited or subtle in children with myopia up to the moderate stage, or that such abnormalities are not adequately captured by the OCTA-derived metrics used here (7, 9, 26, 30–33).

The correlation analyses further support the predominance of structural change over measurable perfusion change. OCT-derived structural parameters showed clear positive associations with SER and negative associations with AL, whereas OCTA-derived perfusion biomarkers showed only weak or selective correlations. Among the structural parameters, HLT showed particularly consistent associations with refractive and axial indices, supporting its potential value as an imaging marker of early choroidal remodeling in childhood myopia. In contrast, the weak correlations between OCT-derived and OCTA-derived metrics suggest limited coupling between structural remodeling and measurable perfusion change within the refractive range examined in this study. This general pattern is consistent with previous pediatric studies showing stronger and more reproducible associations for structural choroidal measurements than for OCTA-derived perfusion biomarkers (9, 12, 19, 21, 26, 29, 31, 34).

These findings may have practical implications. First, OCT-derived structural parameters, particularly full-thickness choroidal thickness, Haller layer thickness, luminal area, and total choroidal area, may be more informative than OCTA-derived perfusion biomarkers for detecting and monitoring early myopic choroidal remodeling in children (6, 12, 21, 23, 26, 29). Second, combined evaluation of full-thickness and sublayer parameters may provide more detailed information than global choroidal thickness alone, because it helps localize the structural compartment most affected during early myopia (22, 23, 35).

This study has several limitations. First, it was a single-center, retrospective cross-sectional study with a modest sample size, which limits generalizability and precludes causal inference. Second, the cohort was restricted to school-aged children from one region, and the findings may not be directly generalizable to populations with different age, ethnic, or refractive characteristics. Third, OCTA-derived perfusion measurements are sensitive to image quality, segmentation strategy, and slab definition, which may affect reproducibility and comparability across studies (7, 16, 25, 31). Fourth, axial-length correction for ocular magnification was not applied to transverse scale-dependent OCT and OCTA parameters. Although this is unlikely to have materially influenced axial thickness measurements, it may have affected area-based and perfusion-related metrics (36, 37). Finally, environmental and familial covariates were not included in the analysis. Larger multicenter longitudinal studies with standardized imaging protocols are needed to clarify the temporal relationship between choroidal remodeling and myopia progression.

## 5 Conclusion

In children with low to moderate myopia, choroidal changes were characterized mainly by structural remodeling, including thinning of the total choroid, predominant involvement of Haller’s layer, and reductions in luminal and total choroidal area. By contrast, OCTA-derived perfusion parameters did not show clear between-group differences. These findings suggest that structural OCT-based choroidal markers may be more informative than OCTA perfusion metrics for detecting early choroidal changes associated with childhood myopia.

## Data Availability

The original contributions presented in the study are included in the article/Supplementary material, further inquiries can be directed to the corresponding author.
