# Supplementary material for: Choroidal structural and perfusion characteristics across refractive groups in children
Source: Front Med (Lausanne). 2026 Jun 10;13:1861658. doi: 10.3389/fmed.2026.1861658 (PMC13290631; doi:10.3389/fmed.2026.1861658)
Supplement: Supplementary file 1 [file Data_Sheet_1.docx]

Supplementary Table 1 ：Appendix Table 1 Comparison of OCT/OCTA image signal quality (dB) among the three groups

| Variables | Total (n = 140) | Emmetropia group (n = 30) | low myopia group (n = 70) | Moderate myopia group (n = 40) | F | P |
| --- | --- | --- | --- | --- | --- | --- |
| OCT_quality | 34.91 ± 4.55 | 35.97 ± 4.52 | 34.34 ± 4.51 | 35.1 ± 4.58 | 1.396 | 0.251 |
| OCTA_quality | 34.89 ± 3.05 | 35.77 ± 3.17 | 34.63 ± 2.88 | 34.67 ± 3.2 | 1.613 | 0.203 |
